# IntroMap: a signal analysis based method for the detection of genomic introgressions

**DOI:** 10.1186/s12863-017-0568-5

**Published:** 2017-12-04

**Authors:** Daniel J. Shea, Motoki Shimizu, Namiko Nishida, Eigo Fukai, Takashi Abe, Ryo Fujimoto, Keiichi Okazaki

**Affiliations:** 1 Laboratory of Plant Breeding, Graduate School of Science and Technology, Niigata University, Ikarashi-ninocho, Niigata, 950-2181 Japan; 2 Iwate Biotechnology Research Center, Narita, Kitakami, 024-0003 Japan; 3 Graduate School of Agricultural Science, Kobe University, Rokkodai, Nada-ku, Kobe, 657-8501 Japan; 4 Department of Computer Science, Graduate School of Science and Technology, Niigata University, Ikarashi-ninocho, Niigata, 950-2181 Japan

**Keywords:** Plant breeding, Interspecific hybridization, Introgression, Signal analysis

## Abstract

**Background:**

Breeding programs often rely on marker-assisted tests or variant calling of next generation sequence (NGS) data to identify regions of genomic introgression arising from the hybridization of two plant species. In this paper we present IntroMap, a bioinformatics pipeline for the screening of candidate plants through the application of signal processing techniques to NGS data, using alignment to a reference genome sequence (annotation is not required) that shares homology with the recurrent parental cultivar, and without the need for *de novo* assembly of the read data or variant calling.

**Results:**

We show the accurate identification of introgressed genomic regions using both *in silico* simulated genomes, and a hybridized cultivar data set using our pipeline. Additionally we show, through targeted marker-based assays, validation of the IntroMap predicted regions for the hybrid cultivar.

**Conclusions:**

This approach can be used to automate the screening of large populations, reducing the time and labor required, and can improve the accuracy of the detection of introgressed regions in comparison to a marker-based approach. In contrast to other approaches that generally rely upon a variant calling step, our method achieves accurate identification of introgressed regions without variant calling, relying solely upon alignment.

**Electronic supplementary material:**

The online version of this article (doi:10.1186/s12863-017-0568-5) contains supplementary material, which is available to authorized users.

## Background

The application of directed introgressive hybridization allows for agronomically important crops to integrate genes from closely related species and serves as an important method for the introduction of beneficial traits from one species of crop cultivar to another [1]. In agricultural and biological research, introgressive hybridzation serves as a useful methodology for the creation of hybridized plant lines, enabling the researcher to examine gene function and molecular pathway interactions of a target gene in a new genetic background [2–4]. The current methodology of marker-assisted selection relies upon the design and analysis of a large number of genomic markers, and requires that genotyping markers be designed for the isolation of both orthologous and paralogous genes present in the parental genomes to accurately discriminate the introgressed gene from the background genome [5, 6]. In many plants, various varieties show distinct differences between their genome and the available reference genome, and often made marker design a difficult and time-consuming process [7–9]. With the advent of the application of NGS technology to marker assisted selection, this process has greatly enhanced the ability to design markers, enabling high-density marker-based screening through the use of restriction fragment length polymorphism (RFLP) and/or amplified fragment length polymorphism (AFLP) markers and simplifying the marker design process [10]. However the screening of cultivars via this method can still be a time-expensive and laborious process when examining large populations, and each generation must be screened in this manner. The ability to rapidly screen large numbers of candidate plants would therefore be beneficial for both commercial plant breeders and researchers alike.

Reduced representation methods, such as restriction association DNA sequencing (RAD-seq) and genotyping by sequencing (GBS) provide methods to genotype germplasm by first applying restriction enzymes to the obtained genomic DNA, followed by library construction and sequencing. The sequences obtained are then aligned to a reference genome, or if no reference is available, aligned internally and single nucleotide polymorphisms (SNPs) are called [11]. This approach provides a method to rapidly screen a large number of samples. In addition, major crops now also have SNP chips available that allow for the same method, but at a further reduced cost.

Statistical based approaches such as ABBA-BABA testing employ Patterson’s *D* statistic to determine if a genome-wide excess of shared derived alleles exists between taxa, but do not reveal which loci show such an excess [12]. Because *D* can be unreliable when effective population size is low, refinements of ABBA-BABA testing have been devised to better identify introgressed loci, but still rely upon variant calling information to assess shared alleles between taxa [13].

The current bioinformatic methods for the detection of genomic introgression that employ NGS, utilize read depths of 10*x*, or more, to discriminate SNPs [14, 15]. Both of the parental cultivars and the progeny are sequenced, and allelic shifts with respect to the parental genomes are determined. However, software currently used to detect introgressed loci relies upon manual identification of regions of introgression within a genome [16].

Studies utilizing statistical analytical techniques concerned with elucidating phylogenetic relationships between evolutionarily divergent organisms perform targeted analysis of highly conserved regions using available reference genomes [17], or whole-genome alignment [18]. Such analysis can provide insight into genetic lineage, and requires either variant calling analysis and the phylogenetic reconstruction of target regions of interest within a genome, or the identification of introgressed regions using variant calling information [17].

By taking advantage of low-cost genomic sequencing, we explored how NGS can further be used to identify the loci of cross-species genomic introgressions, possibly replacing the traditional marker based assays currently used in plant breeding. Towards that goal, we designed IntroMap, a bioinformatic analytical pipeline for the prediction of introgressed regions within a target cultivar’s genome and we include an implementation of our algorithm written in Python using Scientific Python and iPython as a Jupyter notebook [19, 20].

## Methods

### Specification of the algorithm

**Fig. 1:**
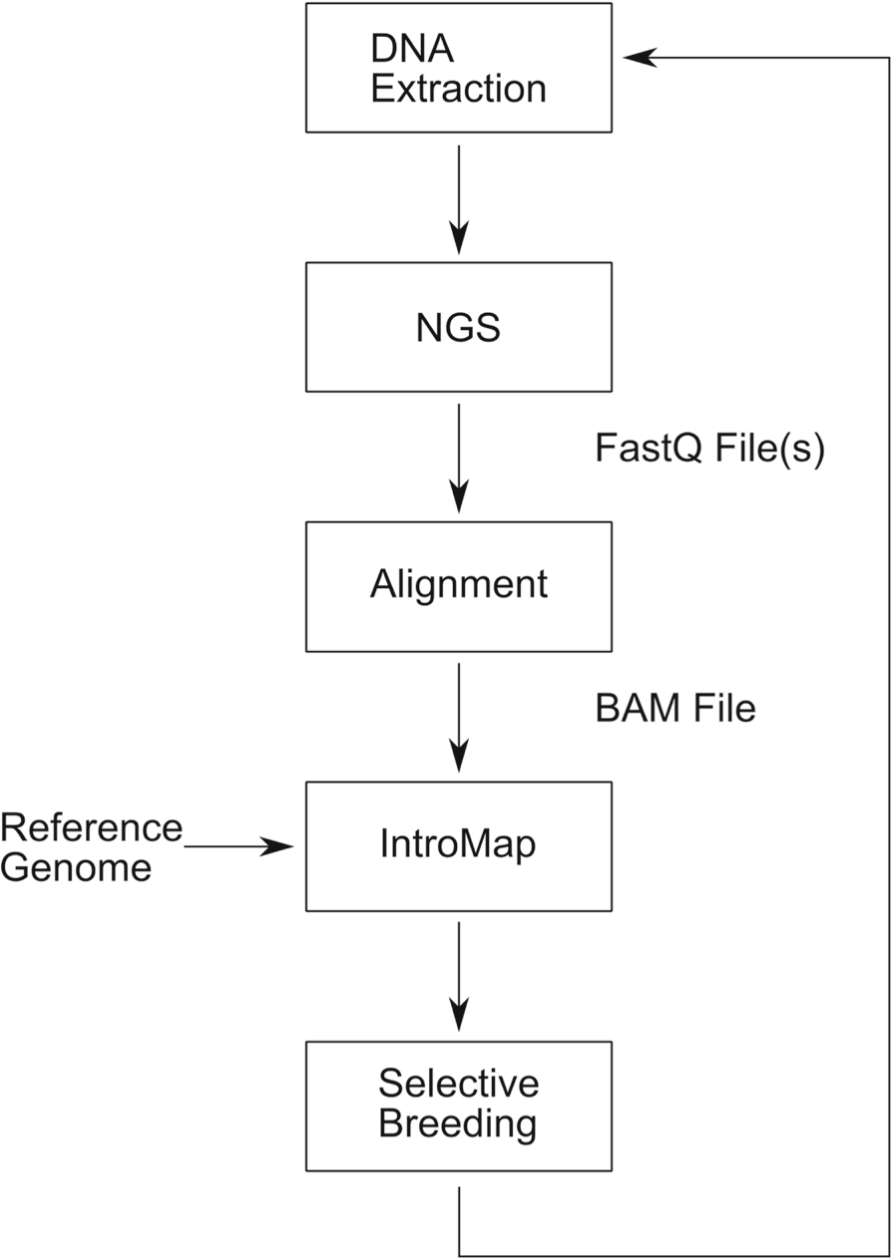
The IntroMap workflow for predicting regions of genomic introgression. Genomic DNA is first extracted and then sequenced. The resulting read data is then aligned to reference producing an binary alignment map. This information is then fed into IntroMap, along with the reference genome to predict regions of genomic introgression with respect to the parental reference

IntroMap requires two inputs, a fasta-formatted file containing the reference sequence genome, and a BAM (Binary SAM) formatted alignment file (created by aligning the NGS reads to the reference using bowtie2), for the prediction of introgressed regions within the alignment. An illustration of the overall work-flow for the application of IntroMap is outlined in Fig. 1.

**Fig. 2:**
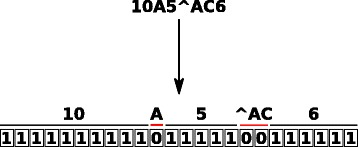
Illustration of how an MD tag is converted to the binary vector $\vec {v}_{i}$, showing matches, mismatches, and an indel

IntroMap first determines a score for each nucleotide position in the reference genome by parsing the MD tags present in each alignment record of the BAM file. The MD tag details each match, mismatch, or deletion for each nucleotide position in the aligned read. The information contained in the MD tag regarding the alignment is represented as a binary vector $\vec {v}_{i}$, where *i*={1…*n*} and *n*= the total number of reads supplied, describing a match (represented by a 1) or a mismatch/indel (represented by a 0) for all base-pair positions along the aligned read (Fig. 2).

After converting each aligned read’s MD tag into a binary vector, IntroMap then constructs a sparse matrix *C*_*d*,*l*_, where: *D*=the maximum read depth, *d*={1…*D*}, *L*_*c*_=the total length of the chromosome *c* in nucleotides, and *l*={1…*L*_*c*_}, such that the values represented by each $\vec {v}_{i}$ are contained in *C*_*d*,*l*_ at their corresponding start coordinates with respect to the reference genome. Regions of the genome where no reads have been aligned are represented by a score of 0.

This is determined by first analyzing the supplied fasta reference file, to determine the lengths of each chromosome *L*_*c*_ present in the reference genome, where *c*={1…the total number of chromosomes in the reference genome }. The mean values for all columns in matrix *C*_*d*,*l*_ are then computed, yielding a vector $\vec {s}_{c}$ that constitutes the per-base calling scores for the overall alignment of that chromosome at each nucleotide position, with coordinates relative to the reference sequence.

Next, IntroMap performs a convolution between $\vec {s}_{c}$ and a vector $\vec {1}_{w}$, whose values are all 1 and whose length *w* defines the window size of the low-pass filter. The convolution acts as a low-pass filter, removing high frequency noise via the averaging of the per-base scores at each nucleotide position with the surrounding per-base scores within a window of size *w*. The resultant vector, $\vec {s^{\prime }}_{c}$, whose length is defined as $||\vec {s^{\prime }}_{c}||=L_{c}-w-1$, is then further processed by a locally weighted linear regression fit function *F*. The fitted signal, $h_{c}=F(\vec {s^{\prime }}_{c})$, contains points along a fitted polynomial curve, and is representative of the overall homology at each position in chromosome *c* between the sequenced cultivar and the reference sequence.

In general, the reference chosen for alignment should share a high degree of homology to that of the genetic parent in order to recover a meaningful signal from the alignment. Each position in *h*_*c*_ contains a real value between [0,1] where 0 is representative of no homology and 1 represents perfect homology. Reconstruction of *h*_*c*_, such that all values were equal to 1, would therefore imply that the aligned reads share perfect homology with the reference genome. Likewise, an *h*_*c*_ signal such that all values were equal to 0 would mean that no reads were aligned, indicating no shared homology between the sequenced cultivar and the reference genome. Application of a low-pass filter acts to suppress the influence of SNPs between the sequenced genome and the reference genome on the overall values that comprise *h*_*c*_. However, large structural variations in homology between the aligned sequence and the reference remain present within the signal.

IntroMap generates a plot of *h*_*c*_ to visualize the shared homology between the seed-parent’s background chromosome *c*_*parent*_ and the hybridized plant’s chromosome *c*_*child*_. We therefore reasoned that regions of the signal that show significant divergence with respect to the parental background genome sequence are likely to be of origin from the other parent’s genome and indicative of a genomic introgression. While any introgressed, syntenic, orthologous gene’s exons will be relatively well-conserved, introns and intergenic regions should show evolutionary-derived sequence divergence. Therefore, the scores of any alignments at these locations will tend towards 0, due to the higher overall number of mismatches and/or deletions. Furthermore, regions that have been deleted from the background genome due to an introgression should also have fewer mapped reads to the reference, resulting in lower overall scores than the highly homologous regions of the background genome mapped back to the reference.

Once *h*_*c*_ has been computed, a threshold function *T*(*h*_*c*_,*t*) is applied to call predicted regions of genomic introgression. The signal *h*_*c*_ is scanned at each position for regions where the score *h*_*c*,*l*_ drops below the threshold value *t* (*h*_*c*,*l*_<*t*), thus marking the beginning of a predicted introgressed region. Likewise, any subsequent rise back above threshold *t* (*h*_*c*,*l*_≥*t*) is marked as the end of the introgression. The region’s coordinates with respect to the reference sequence are then output, along with the graph, showing the computed *h*_*c*_ for each chromosome *c* present in the reference genome.

**Fig. 3:**
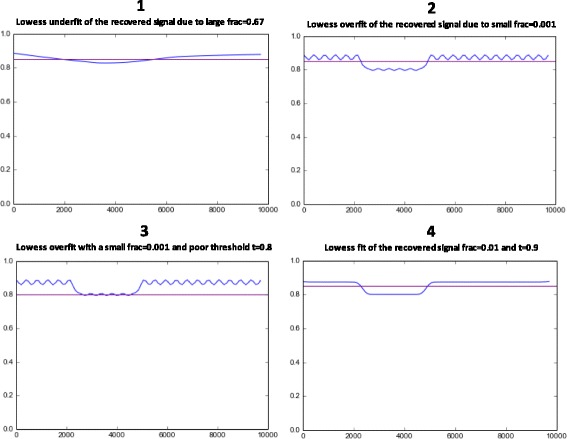
Results of applying three different LOWESS fits to the low-pass processed signal in Fig. 4. Illustrating (**1**) an under-fitting, (**2**) over-fitting, (**3**) over-fitting with a poor choice of threshold, (**4**) and an appropriate fit of the input signal, respectively

The example plots in Fig. 3 illustrate the effects of using various LOWESS fit parameter values (hereafter referred to as *frac*) with a thresholding function *T*(*h*_*c*_,*t*), and where the value of the threshold function’s *t* parameter is also varied. The first plot illustrates the effect of too large a value for the fitting parameter *frac*, resulting in under-fitting of the signal, causing a loss of information of the aligned sequences homology with respect to reference. While IntroMap still detects a large drop in overall homology, it greatly over-estimates the size of the introgressed region. The second plot shows the results of over-fitting to the signal by using too small of a fit parameter value. Here, IntroMap erroneously assigns meaning to local noise in the signal. In this example, it has little effect. However, the next plot illustrates that a poor choice in fit parameter, coupled with a poorly chosen threshold value, may result in false positive introgression predictions for several small regions. The final plot shows typical results when applying appropriate LOWESS fit and threshold parameters (*frac*, *t*). Therefore, it is of some importance to tune the fit parameter and threshold values of IntroMap, prior to conducting large-scale screening. We therefore will next describe the effects of parameter selection and the procedure for parameter tuning by receiver operating characteristic (ROC) analysis.

**Fig. 4:**
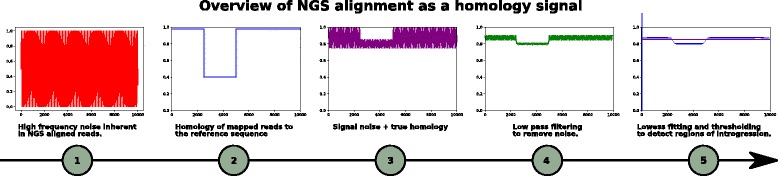
Application of a low-pass filter to suppress high frequency noise. We then further improve upon this by applying locally weighted linear regression fitting (LOWESS) to reduce local minor variations that arise, as in the case of the divergence of non-coding regions of the genome. Finally, a thresholding function is applied to discriminate between the introgressed regions and the native genomic background

While NGS technologies inherently may introduce noise due to errors in sequencing, such errors may be first reduced/removed through quality control of the sequence data prior to alignment. Additionally, some further noise is introduced due to minor differences in the homology of the reference and the parental background genome, as in the case of SNPs and minor divergences of intronic and non-coding/non-conserved regions. To account for this, we first apply a low-pass filter to suppress high frequency oscillations in the homology signal. Small gaps in coverage are more likely indicative of poor coverage at CpG islands, promoter and 5 ^′^-UTR regions of the genome and likely to be homologous to the reference due to their shorter lengths, not as a result of chromosomal introgression [21]. Therefore, we treat such regions as homologous to the background genome through locally weighted linear regression. However, in the case of large unmapped or poorly mapped regions, a lack of coverage has more likely arisen from large-scale structural differences in the genome due to genomic introgression. After low-pass filtering and LOWESS fitting, a threshold function is then applied. This acts as a binary classifier that distinguishes introgressed regions by quantifying how much a signal’s homology must deviate from the reference sequence before an introgression is called.

Note, that the resulting output signal after low-pass filtering results in a slightly truncated number of data points, due to edge effects from applying a convolution that calculates mean values using a sliding window, with respect to the overall signal (Fig. 4). The original signal is 10 kbp in length and the resulting filtered signal is 9701 bp in length in Fig. 4. The resultant signal length is the length of the original signal minus the size of the window of the convolution, minus 1. For short alignments, edge effects may pose an issue. However, in the case of chromosome-scale sequences, where the average length of a chromosome is 25 Mbp in *Brassica rapa*, the application of a window of size 1 kbp accounts for a reduction in overall signal length of the output on the order of 1×10^−5^ and, as we will show, has no effect on our results. Additionally, it should be noted that for cases of copy-number variation (CNV) where repeated sequences may not be present in the reference genome, one drawback of this approach is that IntroMap can not detect such CNVs. Likewise, large indels that exceed the size identified within an aligned read can not be identified in this way.

### Effect of parameter choices for *frac*

The LOWESS fitting function employed by IntroMap is documented in detail in the Python statsmodels manual [22]. Here, we briefly discuss the two parameters utilized for the tuning of the software to better fit a particular genomic data set when using IntroMap. The fit function defines its argument *frac* as the *frac*tion of the data used when estimating each y-value, with valid values in the range [0,1]. The y-value of the plot is our scoring metric, representing homology with respect to the reference genome. As *frac* approaches 1 the fitted function is less-sharply peaked, due to its reliance on base-calling scores of nucleotide positions more distant in the genomic sequence than the nucleotide position being fitted. Likewise, lower values of *frac* result in more-sharply peaked fits, due in part to their reliance on closer nucleotide’s score values. Therefore, we may consider *frac* to represent a window from which data is drawn from, for the purpose of fitting a given nucleotide position’s score and whose size is defined as the value of *frac* multiplied by the length of the reference chromosome *L*_*c*_ (i.e. - fitWindowSize =*f* *r* *a* *c*×*l*_*c*_) for the *h*_*c*_ signal being fitted.

Thus, the choice of *frac* is determined, in part, by the taxa being analyzed, due to the effect of *frac*, and the subsequent change to fitWindowSize, on the run-time of the LOWESS fitting step of IntroMap. Attempting to apply large values results in increases to the run-time on the order of *O*(*f* *i* *t* *W* *i* *n* *d* *o* *w* *S* *i* *z* *e*×*L*_*c*_). To illustrate, the mean length of a chromosome in the *B. rapa* reference genome is 25.73 Mbp in length, thus choosing *frac = 0.10* results in a windowSize of 2.57 Mbp that is then examined at each coordinate along the *L*_*c*_=25.73 Mbp long chromosome, resulting in greatly increased run-times in comparison to smaller values of *frac*.

### Effect of parameter choices for threshold *t*

**Fig. 5:**
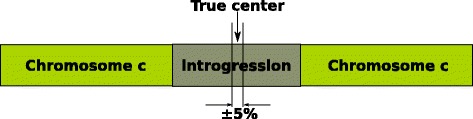
Illustration of the definition of the *loci accuracy* metric. In cases where IntroMap’s predicted center of an introgressed region lie within ±5*%* of the true-center nucleotide position, we record IntroMap as having accurately predicted the center of the introgressed region

The second parameter is the threshold value, represented by *t*. *T*(*h*_*c*_,*t*) is the final function applied to *h*_*c*_, and comprises the binary classifier responsible for predicting if a given region constitutes a genomic introgression. When the score in *h*_*c*_ drops below threshold *t*, IntroMap proceeds to scan *h*_*c*_ until the score rises back above threshold *t*. The region between the two threshold-crossing values is then marked as a predicted region of genomic introgression. Varying the value of *t* has several effects. As *t* is increased towards 1, the likelihood of false positives (FP) increases, particularly if there are gaps in the coverage sufficient to cause local drops in the scores for that region in *h*_*c*_. The threshold value also has an effect on the size of the predicted introgressed regions, as the predicted region’s size will increase as a function of the slopes of the *h*_*c*_ signal at each of the two crossing points at *y*=*t*. Therefore, choice of *t* may perturb the predicted centers of the introgressed regions if the slopes of the lines crossing the threshold value on either side of the region are markedly different. To account for this effect during our analyses, we also compared the center coordinates of predicted regions with the known-center coordinates of the introgressions in our in silico simulated genomes, and recorded when the predicted center and the actual center varied by more than 5*%* of each other. We named this metric *loci accuracy*, to measure what effect varying *t* had on the prediction accuracy of an introgressed region. For example, a loci accuracy value of 75*%* may be interpreted as follows, the correct prediction of 75*%* of all true-positive (TP) introgressed region’s center loci within ± 5*%* of the true centers (Fig. 5).

Furthermore, the selection values of *t*, like *frac*, are also taxa dependent. However in this case, it is not the length of the chromosomes within the genome that are determining factors in parameter selection, but the homology of the recurrent parent’s genome to that of the reference genome. The upper limit on the appropriate choice of *t* is determined by the genetic distance of the recurrent parent to the reference sequence. Selection of *t* as *t*→1 results in an increase in the false positive rate, due to the variations present between the reference and the recurrent parent. Therefore, the selection of *t* should be determined by parameter tuning as described below.

### Receiver operating characteristic (ROC) analysis for selection of *frac* and *t*

The ROC curve represents the sensitivity of a binary classifier as a function of 1−specificity [23]. For the purposes of the prediction of genomic introgression, positive predictive value (PPV) indicates the probability that in the case of a positive call for the existence of a genomic introgression, the genome actually contains the specified introgressed region from the paternal genome. Likewise, negative predictive value (NPV) indicates the probability that in the case of a negative call, that is to say, the absence of a genomic introgression, the genome actually contains genomic material from the maternal genome at the specified region. The function *T*(*h*_*c*_,*t*) is the binary classification function of the IntroMap algorithm, and is affected by the LOWESS fitting function parameter *frac*, therefore when aligning to a reference genome that is highly homologous to the recurrent parent, parameter selection by ROC analysis for the parameters *t* and *frac* is performed.

Using these definitions, we conducted a ROC analysis of the IntroMap software for three in silico simulated genomes to determine acceptable parameters for detecting introgressions of *B. oleracea* derived genomic DNA (gDNA) in a *B. rapa* genomic background.

### In silico genome simulation for parameter selection

To determine the appropriate parameters for calculating *h*_*c*_, and to establish an accurate threshold value *t* for the calling of introgressed regions, we processed the alignments of the three simulated genomes across different values for both the threshold value *t*={0.85,0.90,0.95}, and the LOWESS fitting function’s value *f* *r* *a* *c*={0.05,0.01} on a 12-core Intel Core i7-5930K CPU running at 3.50 GHz with 64 GB of memory. The selected values of threshold *t* were chosen in 5% increments centered around a 90% overall homology between the *B. rapa* reference genome, and recurrent parent *B. rapa* cultivar CR Kanki. Selection of *t* above 95% results in an unacceptable increase of false positives, and selection below 80% results in an unacceptable increase of false negatives. This is due to the shared syntenic blocks of the parental *B. rapa* and *B. oleracea* genomes that resulted from a whole genome triplication (WGT) event and is present in all *Brassicas* [24]. Because the choice of *frac* values are dependent upon the size of the reference genome chromosome lengths being analyzed, our value selection was determined by the computational requirements that are incurred upon performing a locally weighted linear regression for a *h*_*c*_ vector whose length, *L*_*c*_, is equal to that of the entire chromosome. Therefore, we chose two values for *frac*, 0.05 and 0.01, that would provide us with six possible parameter combinations of (*f* *r* *a* *c*,*t*) for tuning by ROC analysis, without incurring an unacceptable run-time penalty due to the window size of the LOWESS fitting step.

In silico simulated genomes were constructed using the known syntenic regions between the published reference genomes for *Brassica oleracea* var. *capitata* and *Brassica rapa* var. *Chiifu-401*. The reference genomes, along with the known syntenic regions were downloaded from Bolbase and BRAD, respectively [25, 26]. A program was then developed in Python, and made available as part of the IntroMap package, to construct three simulated genomes. The simulated genomes are comprised of a *Brassica rapa* genomic background, signifying the maternal seed-parent, and *Brassica oleracea*, representing the paternal parent species. The recurrent parent was selected as the reference to simulate the lineage of the #174-12-26 line whose recurrent parent is a *B. rapa* cultivar. The #174-12-26 line is described in more detail below. The creation of 3 simulated genomes and analysis of the reverse-cross (using *B. oleracea* as the recurrent parent) is provided as Additional file 1: Table S8 through Table S13.

To create the simulated genome files containing known introgressed regions necessary for parameter tuning of IntroMap, the utility *make SimulatedHybrids* was written. The *makeSimulatedHybrids* program randomly replaces segments of the supplied maternal reference genome with segments from the supplied paternal reference genome, according to a list of known syntenic regions that is supplied in a third input file. The psuedo-random number generator, used to simulate random introgressions of known syntenic regions, is made repeatable by specifying the same seed value at run-time. The program also generates a log of the arguments given to it at run-time along with the syntenic regions it has chosen to replace, thereby providing a clear audit trail of the actions taken to create any simulated genomes.

Next, to simulate NGS data, derived from our simulated hybridized genomes, wgsim [27] was applied against the simulated sequence files, generating 50 million paired-end reads that are 75 bp in length, with end-to-end read sizes of 500 bp. The diploid model of read generation was employed in wgsim and the reads modeled to simulate sequencing errors, indels, and SNPs using the default configuration parameters.

Read sets for 3 simulated hybridized genomes were then aligned to the published reference sequence for *Brassica rapa* var. *Chiifu-401* using bowtie2 (version 2.2.6, 64-bit) [28]. The following bowtie2 options were applied during the alignment –no-mixed, –no-discordant, –no-contain, –no-overlap, and –no-unal. Full descriptions of these options are available in the bowtie2 manual. The subsequent SAM output was then converted to BAM format and sorted with samtools [29] by genomic coordinates. After sorting, duplicates were removed by samtools rmdup, generating the final set of BAM files supplied as inputs to IntroMap for analysis.

We then ran IntroMap against the three simulated genomes for varying values of *frac* and *t*, recorded the TPR, FPR, PPV, NPV, and loci accuracy. Then, a ROC plot was created in order to determine suitable parameters for the detection of introgressions of *Brassica oleracea* genomic content in a *Brassica rapa* genomic background.

### Application of IntroMap against the #174-12-26 line

To next test the IntroMap software against actual genomic sequencing data, we utilized a hybrid introgressed plant line, hereafter referred to as #174-12-26. In the *Brassica* genus, *B. rapa*, *B. nigra*, and *B. oleracea* are referred to as the A, B and C genomes, respectively, with the allotetraploid *B. napus* being derived from the interspecific hybridization of the A and C genomes [30]. Unlike *B. napus*, which contains both the A and C genomes (AACC), the #174-12-26 line is derived from a cross between a doubled haploid commercial cultivar *Brassica oleracea* Reiho P01, and a *Brassica rapa* commercial Chinese cabbage cultivar CR Kanki. The #174-12-26 line is a BC _3_ *F*_3_ lineage containing introgressed genomic regions from the *B. oleracea* parent in a *B. rapa* genomic background and therefore only contains partial regions of the C genome introgressed to syntenic A genomic chromosomes (AA+C).

### Plant materials and DNA extraction

Plant materials were collected from the #174-12-26 line. Seeds from this line were germinated in pots and grown in long-day (LD) photo-period conditions at 23°. Once the plants had grown to 3-5 leaves in size, whole, true-leaves were harvested and DNA extracted using the CTAB method [31]. Visualization of extracted DNA was carried out by agarose gel electrophoresis to confirm quality of the materials prior to sequencing.

### Next generation sequencing, alignment to *B. rapa* var. *Chiifu-401* reference, and IntroMap analysis

Extracted DNA was then sequenced on an Illumina NextSeq500, generating on-average 26 million 75 bp paired-end reads per lane. The sequence reads (fastq) were generated from bcl2fastq2 Conversion Software (version v2.16.0). At the same time, adapters were removed and a quality filter was applied to keep reads where 80 percent of the bases had a PHRED quality score of 20 or higher (-q20 -p80). After adapter trimming and quality filtering, the filtered reads were again examined by FastQC (version v0.11.5) and determined to be suitable for alignment to the reference genome and the resulting kept-reads were aligned in the same manner as the simulated data sets to the published reference sequence for *Brassica rapa* var. *Chiifu-401*, as described previously. After the alignment of reads obtained from the #174-12-26 line to the *B. rapa* var. *Chiifu-401* reference genome, both depth and breadth of coverage was computed using bedtools genomecov (version v2.26.0) and then the resulting BAM alignment file was then analyzed via our software, IntroMap, to compute the per-base nucleotide calling scores for the aligned reads in relation to the supplied reference genome (as described previously) and the output examined for the detection of regions of genomic introgression.

### DNA marker design and genotyping assays

IntroMap predicted regions were noted and seven DNA markers designed on amplified fragment length polymorphisms (AFLPs), targeted to genes within the predicted regions, to experimentally verify IntroMap’s predictions (Additional file 2: Table S1). To assess the presence of an introgression on chromosome A02, we first genotyped Reiho, CR Kanki, and two plants of the #174-12-26 introgressed line for *Flowering Locus C* (*FLC2*) (Additional file 3: Figure S1). The designed marker accounted for both paralogous and orthologous genes, producing PCR amplicons of unique lengths for each *FLC2* ortholog due to intron-length polymorphisms. For confirmation of the predicted A09 introgression we used AFLP markers for the orthologs Bra039086 and Bol032218 in *B. rapa* and *B. oleracea*, respectively (Additional file 3: Figure S2). DNA was extracted from samples of both of the parental species CR Kanki and Reiho along with the #174-12-26 line, and additional PCR genotyping assays using our other designed markers for A02 and A09 were conducted (Additional file 3: Figures S1 and S2). All PCR reactions were run using Takara EmeraldAMP polymerase and dNTPs Master Mix at 1x concentration, 10 ng of extracted gDNA and primers at 0.5 *μ*M concentration. PCR reactions were run using 98° melting for 10 s, 60° annealing for 30 s or 60 s and 72° extension for 60 s for 35 cycles. The amplicons were then visualized by 2.0%-agarose gel electrophoresis, based upon the expected PCR amplicon sizes and size differences.

### Analysis of the effects of read coverage

To examine the effect that sequencing coverage has on IntroMap, we generated five simulated NGS sequencing runs of the simulated genome 20160409AC-123 using wgsim as previously described. We simulated 75 bp paired-end reads for 50 M, 40 M, 30 M, 20 M, and 10 M total reads, calculated the depth and breadth of coverage for each read-set, and then ran IntroMap using *f* *r* *a* *c*=0.05 and *t*=0.90, assessing TPR, FPR, and loci-accuracy for the 5 runs. We also examined the effect of sequence coverage on run time and memory utilization, to examine the computational performance of the algorithm.

## Results

### IntroMap analysis of in silico simulated genomes

**Fig. 6:**
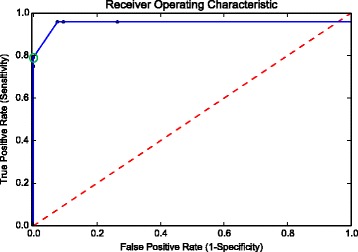
A ROC plot of the results from testing IntroMap across varying LOWESS fitting *frac* and threshold *t* values. The blue solid line is the approximated ROC curve based on the *in silico* simulated genomes. The red-dashed line represents a binary classifier with detection capabilities equal to random chance. The green circle shows the parameters (*f* *r* *a* *c*=0.05,*t*=0.90) for introgression detection of *Brassica oleracea* gDNA in a *Brassica rapa* genomic background before the onset of false positives

The computed genome-wide coverage depth of the alignments for the three simulated data sets were 14.95*x*, 15.02*x*, and 14.90*x* for 20160409AC-1234, 20160409AC-2468, and 20160409AC-36912, respectively. The computed breadth of coverage of the alignments were 94.69*%*, 95.26*%*, 94.16*%* for 20160409AC-1234, 20160409AC-2468, and 20160409AC-36912, respectively. The Additional file 4: Tables S2 through S7 detail the results of running IntroMap against three *in silico* simulated genomes, showing the IntroMap predicted start and end regions of introgression, the actual start and stop coordinates of the introgressions. In the cases of an accurate prediction by IntroMap, the loci accuracy was computed and assessed for accuracy within ± 5*%* of the true center. Table 1 summarizes the results of the analysis, presenting the true-positive rate (TPR), false-positive rate (FPR), positive-predictive value (PPV), negative-predictive value (NPV), and mean loci accuracy for the six test cases across the three simulated genomes. Based on the results of the 18 simulated runs (6 possible [*f* *r* *a* *c*,*t*] parameter combinations × 3 simulated genomes), we determined acceptable parameters for the detection of introgressed *Brassica oleracea* gDNA in a *Brassica rapa* genomic background to be *f* *r* *a* *c*=0.05 and *t*=0.90. These parameters were selected for yielding the greatest computed TPR before the onset of false-positives as seen in the ROC plot (Fig. 6). The run-times of the analysis of the simulated genomes were approximately 35 min per genome, with a peak memory utilization of 55 GB.

**Table 1:** Summary of testing IntroMap across varying LOWESS fitting *frac* and threshold *t* values

frac	t	TPR	FPR	PPV	NPV	Mean loci accuracy
0.05	0.85	0.750	0.000	1.000	0.898	0.667
0.05	0.90	0.792	0.000	1.000	0.914	0.750
0.05	0.95	0.792	0.000	1.000	0.914	0.750
0.01	0.85	0.958	0.094	0.821	0.980	0.958
0.01	0.90	0.958	0.075	0.852	0.980	0.917
0.01	0.95	0.958	0.264	0.622	0.975	0.917

Mean loci accuracy is defined as the percentage of the time that IntroMap’s loci accuracy was within 5*%* of the true center

The tuning of the fit parameters in IntroMap is a recommended procedure, and should be done prior to its use on actual genomic data. The tuning process ensures acceptable discrimination by the binary classifier. Ideally, future versions of this software will incorporate this step in an automated fashion, alleviating the burden of the parameter tuning process placed upon the end-user. On the other hand, manual tuning provides the user with the flexibility of deciding upon an acceptable FPR rate with respect to their intended use-case and experimental design.

### Predicting introgressed genomic regions in the #174-12-26 hybridized line

**Fig. 7:**
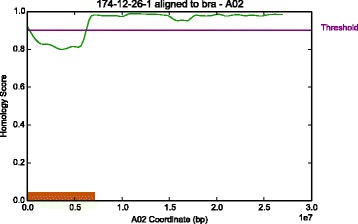
Detected introgression on chromosome A02. IntroMap results for chromosome A02 using parameters (*f* *r* *a* *c*=0.05,*t*=0.90) for introgression detection of *Brassica oleracea* gDNA in a *Brassica rapa* genomic background. The green line is the computed signal *h*_*A*02_ The threshold is indicated by a purple line at *y*=0.90 and labelled accordingly. The orange box shows a detected introgression from 228,453 bp to 6,213,700 bp

**Fig. 8:**
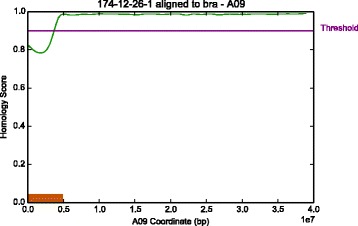
Detected introgression on chromosome A09. IntroMap results for chromosome A09 using parameters (*f* *r* *a* *c*=0.05,*t*=0.90) for introgression detection of *Brassica oleracea* gDNA in a *Brassica rapa* genomic background. The green line is the computed signal *h*_*A*09_ The threshold is indicated by a purple line at *y*=0.90 and labelled accordingly. The orange box shows a detected introgression from 1 bp to 3,703,909 bp

The computed genome-wide coverage of the reads was 18.35*x*, with a breadth of coverage of 90.81*%* of the *B. rapa* genome. IntroMap detected two regions of introgression in the #174-12-26 line. The first region spanned approximately 6 Mbp (228,453 – 6,213,700 bp) on the short-arm of Chromosome A02 (Fig. 7), and the second region detected spanned approximately 3.7 Mbp (1 – 3,703,909 bp) on the short-arm of Chromosome A09 (Fig. 8). Running IntroMap against an alignment of the #174-12-26 reads aligned to the *B. oleracea* reference genome with frac=0.01, and threshold=0.90 determined by performing additional ROC analysis on simulated C genomes containing syntenic A introgressions, yielded results consistent with that of alignment to *B. rapa* (Additional file 3: Figure S3). However, it must be noted that in the reverse predictions, the predicted regions are not as clearly elucidated by IntroMap. This is possibly attributed to the fact that the #174-12-26 line is an A genomic background line and the parental C genome plant (Reiho) is possibly more divergent with respect to its reference than the A parental genome (CR Kanki), resulting in lower homology scores for the C derived genomic regions. Additionally, the majority of reads derived from #174-12-26 sample should be of A genomic origin, and aligning them to a C genome will yield more mismatches and gaps in coverage due to a lower number of aligned reads, thus lowering overall homology scores at each position. This can be seen in the alignment rates of the #174-12-26 reads to the *B. rapa* and *B. oleracea* reference genomes, where alignment to the A genome results in 31.87% of 65,015,089 paired end reads mapping concordantly exactly one time, with an overall alignment rate of 55.77%. On the other hand, the alignment of #174-12-26 reads to the C genome results in only 14.07% of the reads mapping concordantly exactly one time, with an overall alignment rate of 37.19%. In spite of these short-comings, IntroMap alignment against the C genome produces a noticeably inverse response, with respect to outputs derived from alignment to the A genome, suggesting that the introgressed regions are present in the #174-12-26 line at their respective loci.

### Experimental verification of IntroMap’s predicted regions via marker-based assays

The genotyping assays experimentally confirmed the existence of the A02 and A09 predicted regions of introgression showing these regions to be derived from the Reiho genome. The A02 introgression spans approximately from 200 kbp to 6.35 Mbp, using A genome coordinates. The introgressed regions are consistent with the known published syntenic blocks between the *B. rapa* and *B. oleracea* genomes, also referred to as the A and C genomes, respectively, indicating that they are chromosomal loci where recombination events are more likely to occur during hybridization between the two species. The A02 introgression is derived from the R-block, and the A09 introgression appears to be the O-block [32] of the C and A genomes of *Brassica oleracea* and *Brassica rapa*, respectively. Composite figures of the 2% agarose gels showing the genotyping and mapping results for A02 and A09 are shown in the Additional file 3: Figures S1 and S2. The primers used are listed in Additional file 2: Table S1. Chromosomal alignments, depicting the marker locations were also created using Symap [33] from the published reference sequences and are available in the supplied Additional file 3: Figures S1 and S2 for C02/A02 and C09/A09, respectively. The result of our marker assays show that the estimated size and location of the introgressed regions are consistent with IntroMap’s predictions for the #174-12-26 line.

The #174-12-26 line was developed to introgress the *B. oleracea* homolog of *A. thaliana* *FLOWERING LOCUS C* (*BoFLC2*) into *B. rapa* for the characterization of flowering time in response to cold treatment. It should be noted that the previous genome wide marker assays performed during the development of the #174-12-26 line noted the existence of an introgression at A09 in the #174 (BC _2_ *F*_1_) population, however the size of the introgression was unknown and its transmission to subsequent generations was not monitored. Only after the subsequent sequencing and analysis by IntroMap was the A09 locus size and its transmission to #174-12-26 line elucidated and then validated by targeted marker assays, highlighting the utility of the application of NGS technology to plant breeding.

### Read coverage below 10*x* reduces run time but impairs detection

The five simulated NGS read-sets ranged in depth of coverage from 14.95*x* to 2.99*x*, and in breadth of coverage from 94.69*%* to 90.03*%*. The results of each run are shown in Table 2. Alignment rates were consistently 51.4% for the five data sets as shown by the number of aligned reads. Run times and memory scaled roughly linearly with the size of the read sets. Once coverage depth falls below 9x, the TPR of IntroMap appears to significantly affected, as evidenced by the drop in TPR for runs L004 and L005. Interestingly, while the TPR of run L004 fared no better than random chance, the PPV and NPV of the algorithm in this configuration remained at 1.0 and 0.8, respectively, and the mean loci accuracy was unchanged (Additional file 5: Table S14 through S18); indicating that while many introgressed regions may be missed due to an insufficient depth of coverage, the fidelity of the called introgressions remained high. Thus, we concluded that a coverage depth of 10x, or greater, is recommended to improve the detection of introgressions.

**Table 2:** Results of testing IntroMap across various NGS read coverage for the 20160409AC-1234 simulated genome (*f* *r* *a* *c*=0.05,*t*=0.90)

NGS read-set	Coverage depth (x)	Coverage breadth (%)	TPR	FPR	Mean loci accuracy	Run time	Peak memory (GB)	Aligned read pairs
L001	14.95	94.69	0.70	0.00	0.25	35m10s	55	25.65 M
L002	11.97	94.66	0.60	0.00	0.21	34m21s	50	20.53 M
L003	8.98	94.59	0.60	0.00	0.21	22m07s	32	15.40 M
L004	5.99	94.29	0.50	0.00	0.21	15m56s	30	10.27 M
L005	2.99	90.03	0.30	0.00	0.13	9m43s	30	5.14 M

## Discussion

### A novel method for the screening of genomes

We have provided a method for the identification of genome-wide introgressions that employs a signal-analysis-based approach that is capable of identifying and reporting introgressed loci, and we verified the accuracy of our algorithm using both *in silico* simulated genomes and interspecific hybridized genomic data that was verified by marker-based assays. IntroMap does not rely upon variant calling analysis, can readily be automated to screen a large number of plants faster than traditional marker-based methods, and identifies regions of introgression by reporting introgressed loci across the genome in an automated fashion using information obtained by alignment to the reference genome of the recurrent parent. Furthermore, the reference sequence need not be the exact sequence of the parental background genome, provided that the reference sequence applied shares overall structural homology with that of the seed parent, and the reference used for alignment does not need to be annotated.

### Limitations of this method

Our coverage analysis shows that IntroMap is dependent upon a sufficient coverage for the accurate detection of introgressions, similar to variant calling techniques which are reliant upon a sufficient depth of coverage [14]. Additionally, these results indicate that small or highly homologous introgressions may be missed by our method, as one particular 283 kbp syntenic region in the simulated 20140609AC-1234 data set went undetected in all test cases, highlighting a shortcoming of our algorithm to detect small introgresions. Likewise, reliance on the sequence and structure of the reference genome means that CNVs and indels are also missed by our method. However, in spite of these drawbacks, our results indicate that the PPV and NPV of IntroMap are suitable for the screening hybridized progeny, as the detected regions of introgression are accurately deduced.

## Conclusions

### Applications for plant breeding and genetics

It is anticipated that the cost of genomic sequencing will continue to decline in cost [34], thus software able to identify the loci of genomic introgressions is a useful tool for molecular breeding. To assist in the application of molecular breeding to plant hybridization, and the conferral of the favorable traits of one species to another for the purposes of agricultural production, IntroMap may be useful to breeders by helping to identify the loci responsible for a desired agricultural trait. With the continuous improvements made to next-generation sequencing, both in technological capability and price-performance, bioinformatic methods such as IntroMap can provide faster, less laborious, and information-enriched methods for plant breeding research. The application of interspecific hybridization for the conferral of agronomically important traits has a long history and is an enormously important aspect of agricultural research [35–37]. IntroMap provides an additional bioinformatic tool, that employs a novel method, for further studies in this area of research.

## Additional files

Additional file 1 Tables S8 through S13. Each table in the Excel workbook contains results of a run of IntroMap on in silico simulated data using *B. oleracea* as the recurrent parent for a given frac and threshold combination. (XLSX 28 kb)

Additional file 2 Table S1. This spreadsheet contains the primers used for the verification of the #174-12-26 regions of introgression detected by IntroMap analysis. (XLSX 12 kb)

Additional file 3 Supplemental figures. The powerpoint file contains Supplemental figures S1 through S3. These figures show composite images of agarose gel electrophoresis results from the marker-based assays, diagrams describing the location of the markers for chromosomes A02/C02 and A09/C09, and the IntroMap plots for Chromosomes C02 and C09 that resulted from the alignment of #174-12-26 reads to the *B. oleracea* reference genome, followed by analysis of the BAM file via IntroMap. (PPTX 1802 kb)

Additional file 4 Tables S2 through S7. Each table in the Excel workbook contains results of a run of IntroMap on *in silico* simulated data using *B. rapa* as the recurrent parent for a given *frac* and *threshold* combination. (XLSX 34 kb)

Additional file 5 Tables S14 through S18. Each table in the Excel workbook contains results of a run of IntroMap, using *f* *r* *a* *c*=0.05 and *t*=0.90, on the 20160409AC-1234 genome for varying NGS read coverages. (XLSX 16 kb)

## Abbreviations

AFLP: Amplified fragment length polymorphism
FPR: False positive rate
GBS: Genotyping by sequencing
gDNA: genomic DNA
HMM: Hidden Markov Model
NGS: Next Generation Sequencing
NPV: Negative predictive value
PPV: Positive predictive value
RAD-seq: Restriction association DNA sequencing
RFLP: Restriction fragment length polymorphism
ROC: Receiver operating characteristic
SNP: Single nucleotide polymorphism
TPR: True positive rate
VCF: Variant call file
