# Supplementary material for: IntroMap: a signal analysis based method for the detection of genomic introgressions
Source: BMC Genet. 2017 Dec 4;18:101. doi: 10.1186/s12863-017-0568-5 (PMC5716257; doi:10.1186/s12863-017-0568-5)
Supplement: Supplementary file 3 — Supplemental figures. The powerpoint file contains Supplemental figures S1 through S3. These figures show composite images of agarose gel electrophoresis results from the marker-based assays, diagrams describing the location of the markers for chromosomes A02/C02 and A09/C09, and the IntroMap plots for Chromosomes C02 and C09 that resulted from the alignment of #174-12-26 reads to the B. oleracea reference genome, followed by analysis of the BAM file via IntroMap. (PPTX 1802 kb) [file 12863_2017_568_MOESM3_ESM.pptx]

## Slide 1
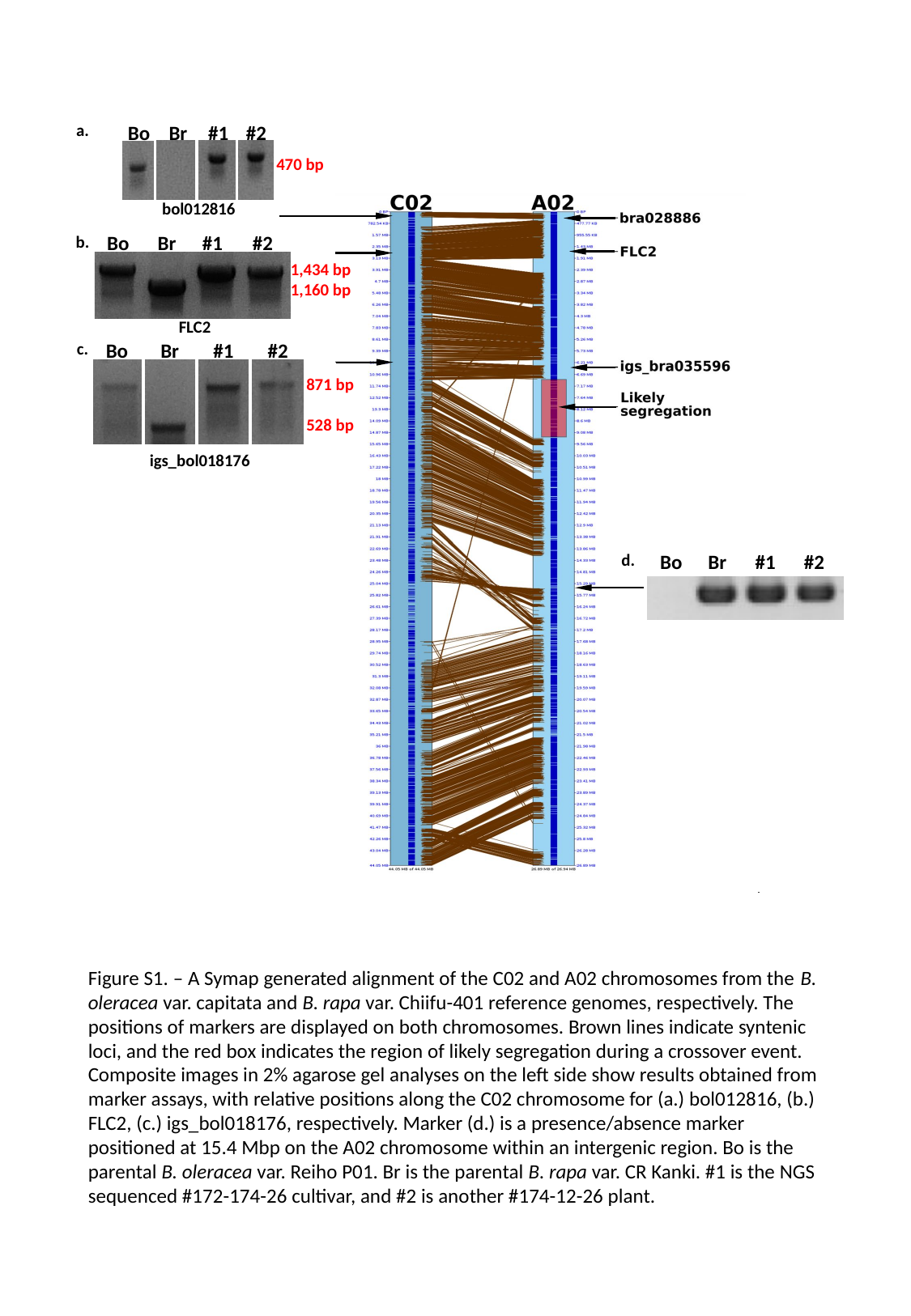

a.
Bo
Br
#1
#2
470 bp
bol012816
Bo
Br
#1
#2
1,434 bp
1,160 bp
b.
FLC2
Bo
Br
#1
#2
871 bp
528 bp
c.
igs_bol018176
Bo
Br
#1
#2
d.
Figure S1. – A Symap generated alignment of the C02 and A02 chromosomes from the B. oleracea var. capitata and B. rapa var. Chiifu-401 reference genomes, respectively. The positions of markers are displayed on both chromosomes. Brown lines indicate syntenic loci, and the red box indicates the region of likely segregation during a crossover event. Composite images in 2% agarose gel analyses on the left side show results obtained from marker assays, with relative positions along the C02 chromosome for (a.) bol012816, (b.) FLC2, (c.) igs_bol018176, respectively. Marker (d.) is a presence/absence marker positioned at 15.4 Mbp on the A02 chromosome within an intergenic region. Bo is the parental B. oleracea var. Reiho P01. Br is the parental B. rapa var. CR Kanki. #1 is the NGS sequenced #172-174-26 cultivar, and #2 is another #174-12-26 plant.

## Slide 2
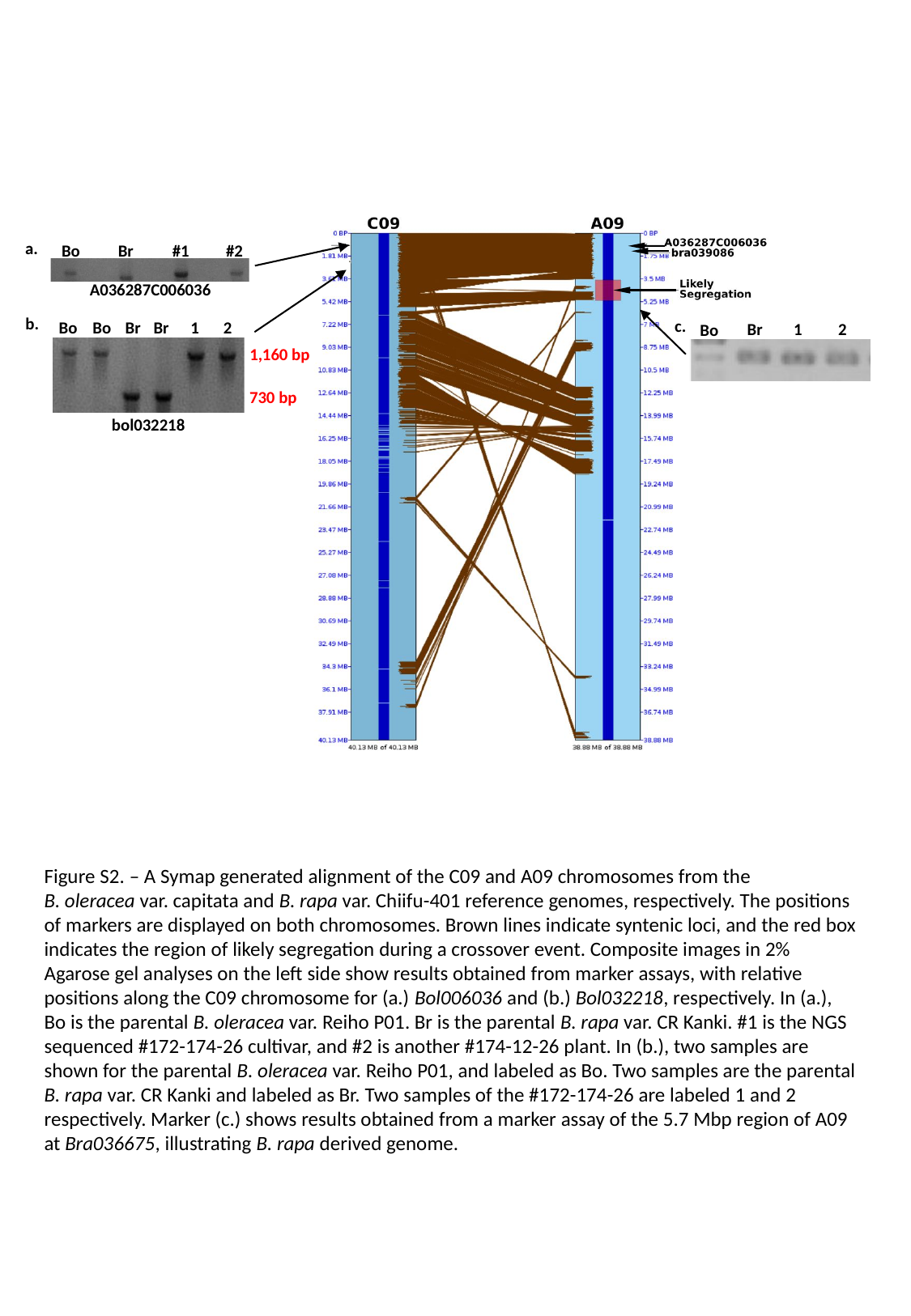

a.
Bo
Br
#1
#2
A036287C006036
b.
c.
Bo
Bo
Br
Br
1
2
1,160 bp
730 bp
1
2
Br
Bo
bol032218
Figure S2. – A Symap generated alignment of the C09 and A09 chromosomes from the
B. oleracea var. capitata and B. rapa var. Chiifu-401 reference genomes, respectively. The positions of markers are displayed on both chromosomes. Brown lines indicate syntenic loci, and the red box indicates the region of likely segregation during a crossover event. Composite images in 2% Agarose gel analyses on the left side show results obtained from marker assays, with relative positions along the C09 chromosome for (a.) Bol006036 and (b.) Bol032218, respectively. In (a.), Bo is the parental B. oleracea var. Reiho P01. Br is the parental B. rapa var. CR Kanki. #1 is the NGS sequenced #172-174-26 cultivar, and #2 is another #174-12-26 plant. In (b.), two samples are shown for the parental B. oleracea var. Reiho P01, and labeled as Bo. Two samples are the parental B. rapa var. CR Kanki and labeled as Br. Two samples of the #172-174-26 are labeled 1 and 2 respectively. Marker (c.) shows results obtained from a marker assay of the 5.7 Mbp region of A09 at Bra036675, illustrating B. rapa derived genome.

## Slide 3
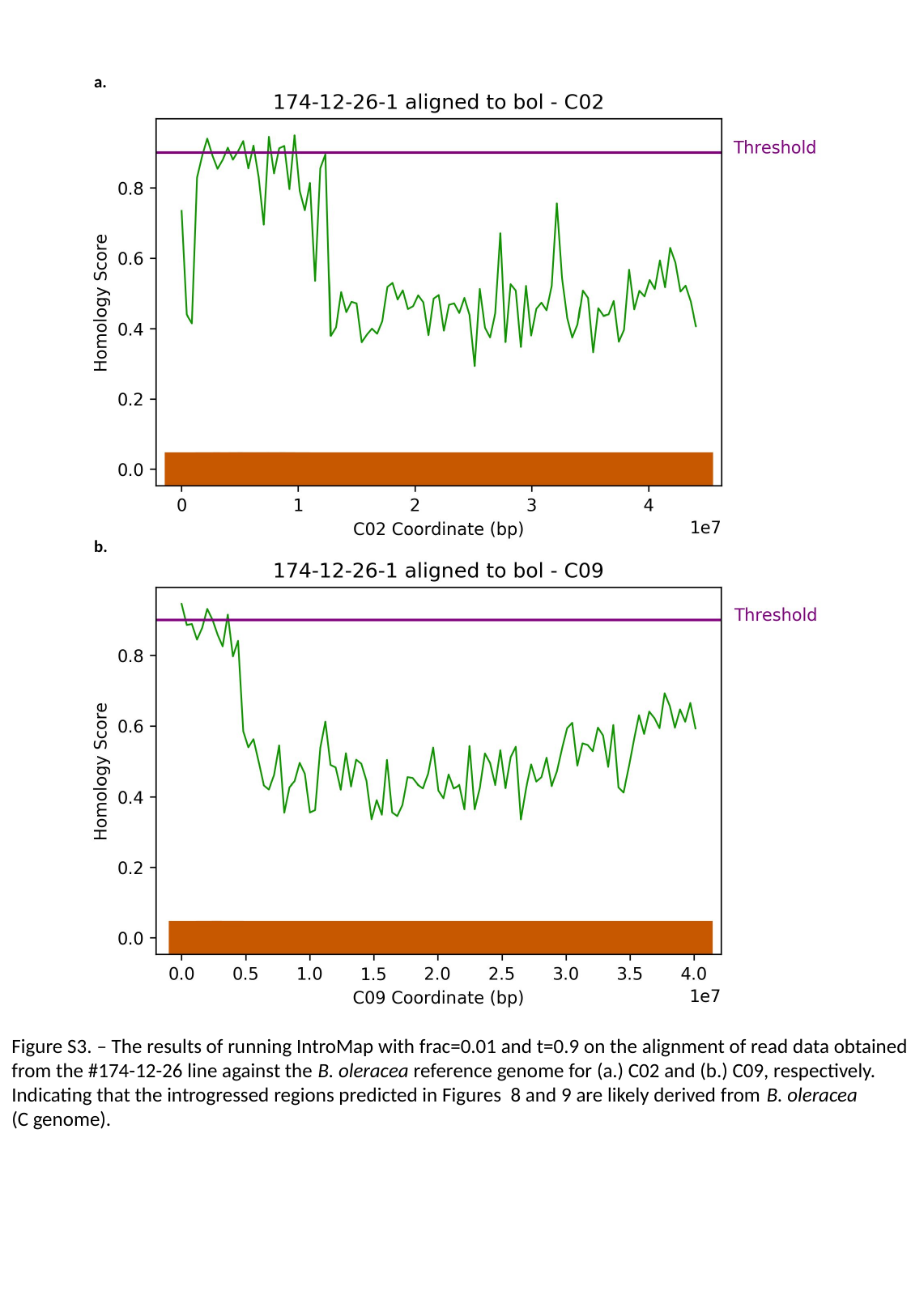

a.
b.
Figure S3. – The results of running IntroMap with frac=0.01 and t=0.9 on the alignment of read data obtained
from the #174-12-26 line against the B. oleracea reference genome for (a.) C02 and (b.) C09, respectively.
Indicating that the introgressed regions predicted in Figures 8 and 9 are likely derived from B. oleracea
(C genome).
